# Integrating perioperative inflammation and pathological invasiveness to predict recurrence after curative colorectal cancer surgery: a multicenter cohort study

**DOI:** 10.3389/fonc.2026.1861588

**Published:** 2026-08-12

**Authors:** Jian Deng, Qiang Gao, Jie Jiao, Baochuan Yang, Xunying Zhang, Jiayong Wang, Jinbo Jiang, Zhi Liu

**Affiliations:** 1 Department of General Surgery, Qilu Hospital of Shandong University, Jinan, Shandong, China; 2 Department of Gastrointestinal Surgery, Central Hospital Affiliatedo Shandong First Medical University, Jinan, Shandong, China; 3 Department of Gastrointestinal Surgery, The Third Affiliated Hospital of Shandong First Medical University, Jinan, Shandong, China; 4 Department of Gastrointestinal Surgery, Jinan Fourth People’s Hospital, Jinan, Shandong, China

**Keywords:** colorectal cancer, external validation, prognostic model, recurrence-free survival, systemic inflammation, tumor-host interaction

## Abstract

**Background:**

Recurrence risk after curative surgery for non-metastatic colorectal cancer (CRC) remains heterogeneous within the same TNM stage. Pathological invasiveness and perioperative systemic inflammation may provide complementary prognostic information, but their combined value and the role of early postoperative inflammatory dynamics remain insufficiently defined. We aimed to develop and externally validate a multicenter prognostic model integrating invasive pathological features with perioperative inflammatory biomarkers to improve recurrence-free survival (RFS) prediction.

**Patients and methods:**

We retrospectively included 1,260 patients with stage I-III colorectal adenocarcinoma who underwent curative-intent resection between January 2017 and March 2023. The development cohort comprised 1,008 consecutive patients from Qilu Hospital of Shandong University, and the external validation cohort comprised 252 patients from three regional hospitals. Data extraction followed a prespecified case-report framework with harmonized definitions for pathology, perioperative laboratory timing, adjuvant treatment, surgical approach, MSI/MMR status, and follow-up. The final Cox model was evaluated against prespecified comparator models, with additional sensitivity analyses adjusting for adjuvant chemotherapy, surgical approach, and MSI status.

**Results:**

The reduced final pathology-inflammation model incorporated eight predictors: histological differentiation, pN stage, extramural venous invasion (EMVI), tumor budding (BD), tumor deposits (TD), preoperative CEA, preoperative systemic immune-inflammation index (preSII), and postoperative day-1 C-reactive protein-to-albumin ratio (postCAR). The final model used 9 effective parameters and 437 recurrence events in the development cohort, corresponding to an events-per-variable ratio of 48.6. Its apparent Harrell C-index was 0.786 in the development cohort and 0.793 in the external validation cohort; bootstrap-based internal validation showed minimal optimism (mean optimism 0.003; optimism-corrected C-index 0.783). Adding adjuvant chemotherapy, surgical approach, and MSI status did not materially change discrimination in either cohort. The development-derived cutoff retained clinically meaningful separation in the external validation cohort, with 3-year sensitivity of 78.0% and specificity of 72.5%.

**Conclusions:**

In this multicenter study, a reduced pathology-inflammation model improved postoperative recurrence prediction beyond conventional clinicopathologic assessment in stage I-III CRC and remained stable after adjustment for newly incorporated treatment, surgical approach, and MSI/MMR information. Because postoperative inflammatory markers may partly reflect operative and early postoperative factors, and because the study remains retrospective, the model should be interpreted as a risk-stratification aid requiring prospective validation before routine clinical implementation.

## Introduction

Colorectal cancer (CRC) remains one of the most commonly diagnosed malignancies worldwide and a major cause of cancer-related mortality (1). Although curative-intent resection is the standard treatment for non-metastatic disease, postoperative recurrence remains a major determinant of long-term outcome (2). Accurate postoperative risk stratification is therefore important for tailoring surveillance intensity and refining individualized management after surgery. Beyond anatomic disease burden, recurrence is increasingly understood as a consequence of interactions among residual tumor cells, invasive pathological phenotype, and the systemic host response.

The 8th edition AJCC TNM staging system remains the foundation of postoperative risk assessment in CRC, yet it does not fully explain why recurrence varies markedly among patients within the same stage group (3). This unmet need has focused attention on pathological features that capture biological aggressiveness beyond anatomic extent. Extramural venous invasion (EMVI), identified radiologically or histopathologically, has been associated with synchronous metastasis and poor oncologic outcomes (4, 5). Tumor budding (BD), a histological marker linked to epithelial-mesenchymal transition, has also been validated as an adverse prognostic factor, particularly in stage II-III CRC (6, 7). Likewise, tumor deposits (TD) reflect invasive and metastatic potential and are recognized as markers of unfavorable prognosis (8). These features describe the tumor-invasive component of recurrence risk, but they do not directly capture the systemic host context in which recurrence develops.

In parallel, increasing evidence indicates that host systemic inflammation contributes to CRC progression and recurrence (9). Composite inflammatory indices such as the systemic immune-inflammation index (SII) and the C-reactive protein-to-albumin ratio (CAR) reflect the interplay among neutrophil- and platelet-driven inflammation, lymphocyte-mediated immune competence, acute-phase activation, and nutritional reserve (10, 11). Among these, CAR has been repeatedly associated with poor survival outcomes in CRC and other solid tumors (12, 13). Emerging data further suggest that perioperative inflammatory dynamics may provide additional prognostic information beyond preoperative measurements alone, because surgery can transiently amplify innate inflammatory pathways and immunosuppressive recovery states. However, early postoperative biomarkers, including postoperative CAR (postCAR), have rarely been incorporated into multivariable prognostic models for postoperative recurrence (14, 15).

Existing prediction tools remain limited in several respects. Most rely primarily on stage or conventional clinicopathological variables and do not simultaneously integrate invasive pathological features with perioperative inflammatory response markers (16, 17). In addition, potentially nonlinear associations between inflammatory biomarkers and oncologic outcomes are often ignored, and the incremental value of newly incorporated host-response biomarkers is seldom formally quantified (18, 19). These limitations highlight the need for a more comprehensive and rigorously validated model that links tumor invasiveness with clinically measurable systemic inflammation in contemporary surgical cohorts.

In this multicenter study, we aimed to develop and externally validate a prognostic model integrating invasive pathological features (BD, TD, and EMVI), preoperative SII, and postoperative CAR for recurrence risk prediction after curative resection of CRC. We further sought to distinguish benchmark comparator models used for incremental context from the reduced final model intended for nomogram construction and clinical application.

## Materials and methods

### Study design and cohorts

This multicenter retrospective cohort study was coordinated by Qilu Hospital of Shandong University and reported in accordance with the TRIPOD statement. The study protocol was approved by the Ethics Committee of Qilu Hospital of Shandong University (approval number: KYLL-2026ZM-1243). The requirement for written informed consent was waived by the ethics committee because of the retrospective design and use of de-identified clinical data. A total of 1,260 patients with histopathologically confirmed colorectal adenocarcinoma who underwent curative-intent resection between January 2017 and March 2023 were included. The development cohort comprised 1,008 consecutive eligible patients treated at Qilu Hospital of Shandong University, and the external validation cohort comprised 252 consecutive eligible patients from three additional participating hospitals. All centers used the same eligibility criteria, variable definitions, and follow-up framework.

Before data pooling, participating centers used a harmonized data dictionary defining eligibility, operative date, recurrence, censoring, laboratory time windows, pathology variables, adjuvant treatment, surgical approach, and molecular-marker categories. Data were abstracted from electronic medical records and pathology reports by trained investigators at each center and were checked centrally for range, coding, and logical consistency. Laboratory tests were obtained from hospital-accredited laboratories at fixed perioperative time points: within 1 week before surgery and on postoperative day 1. Follow-up information was collected from outpatient records, imaging reports, readmission records, and telephone follow-up using a common recurrence definition.

**Figure 1:**
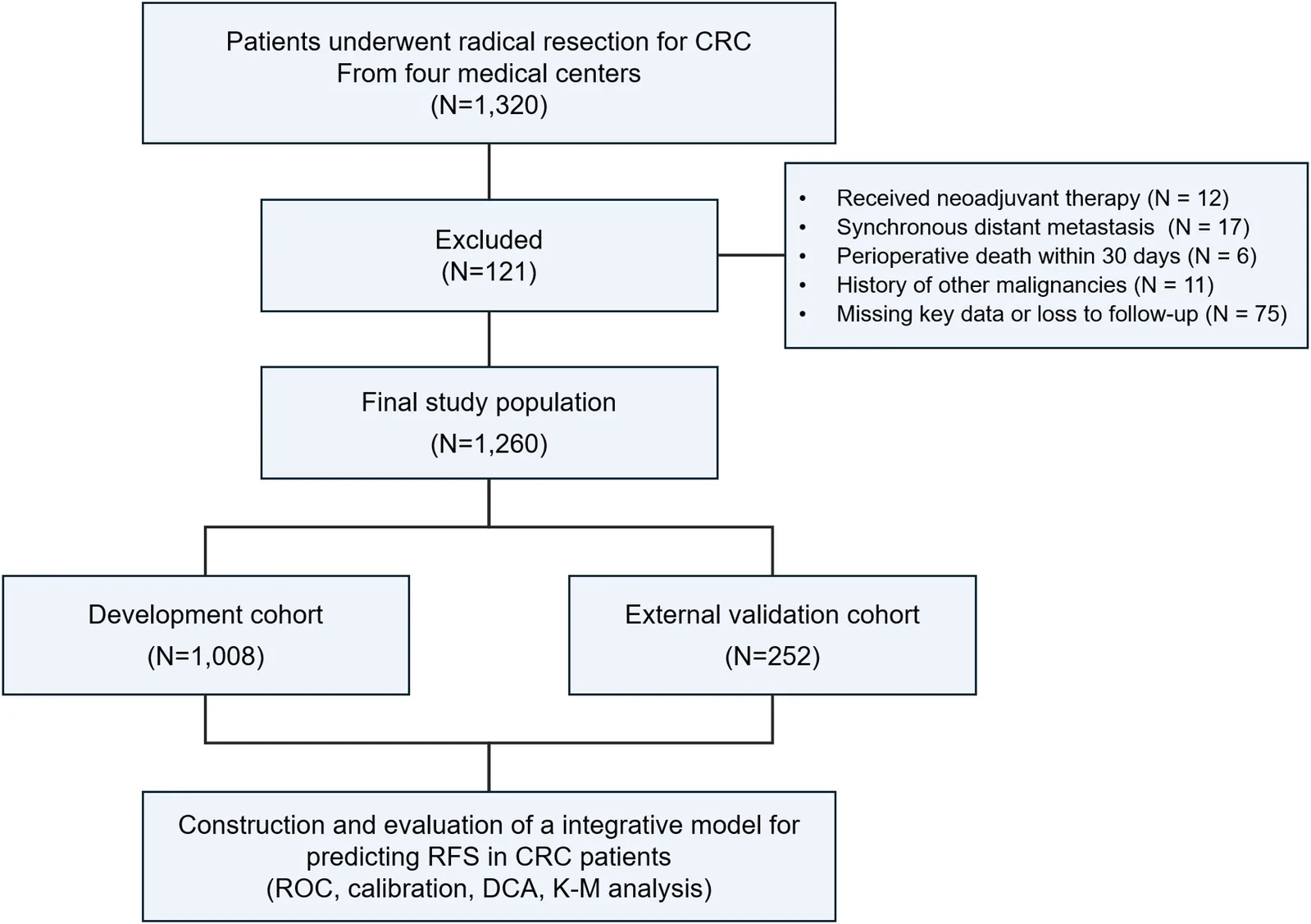
Study flow diagram for multicenter patient selection, cohort assignment, and external validation.

Inclusion criteria were as follows: (1) curative resection with negative margins; (2) availability of clinicopathological data and perioperative laboratory tests obtained within 1 week before surgery and on postoperative day 1; and (3) complete follow-up information for RFS. Exclusion criteria were: (1) neoadjuvant therapy; (2) synchronous distant metastasis; (3) perioperative death within 30 days; (4) a history of other malignancies; and (5) missing key predictors or loss to follow-up. The study flow diagram (**Figure 1**) details patient screening and the number excluded at each step.

Patients with missing required baseline, pathological, perioperative laboratory, recurrence, or follow-up information were excluded at cohort assembly. In the final verified analytic datasets used for model development and external validation, all variables included in the final model and reviewer-requested sensitivity analyses were complete. Missing-data rates for each analytic variable are reported in **Supplementary Table 5**.

### Outcome definition and follow-up

RFS was the prespecified primary endpoint and was defined as the interval from surgery to the first documented local or distant recurrence. Patients without recurrence were censored at the date of last follow-up, and patients who died without a documented recurrence were censored at the date of death under this recurrence-focused endpoint definition. Postoperative surveillance followed institutional protocols aligned with NCCN/ESMO recommendations, with clinical review every 3–6 months during the first 2 years and every 6–12 months thereafter for up to 5 years. Routine assessments included physical examination, serum carcinoembryonic antigen (CEA), and contrast-enhanced computed tomography of the chest, abdomen, and pelvis. Recurrence was confirmed by radiological findings suggestive of malignancy, endoscopic evidence of local relapse, or histopathological confirmation when feasible. The study therefore focused on recurrence risk rather than all-cause disease control, and non-recurrence death was not modeled as a competing event in the primary analysis.

### Predictor collection and definitions

Clinicopathological variables were extracted from institutional electronic medical records using a prespecified data-collection framework. Baseline variables included age, sex, and body mass index (BMI). Tumor-related variables included tumor location, histological differentiation, and pathologic stage according to the 8th edition of the American Joint Committee on Cancer (AJCC) TNM system. Pathological high-risk features, including EMVI, lymphovascular invasion, perineural invasion, tumor deposits, and tumor budding, were assessed according to routine pathology review at each center. Circumferential resection margin involvement was defined as a distance of ≤1 mm between the tumor and mesorectal fascia. Detailed data on adjuvant chemotherapy, postoperative radiotherapy, targeted therapy, immunotherapy, and molecular markers were not uniformly available across all centers and therefore were not included in the main prediction framework.

Pathological variables were abstracted from formal pathology reports generated according to routine institutional gastrointestinal pathology practice and harmonized during data extraction according to AJCC 8th edition staging definitions. Tumor budding was coded according to reported grade categories, and EMVI, lymphovascular invasion, perineural invasion, tumor deposits, and CRM status were coded using prespecified binary definitions. Because no centralized pathology rereview was performed, inter-center interpretive variability for EMVI, tumor budding, and tumor deposits was considered in the interpretation of model performance and is explicitly acknowledged as a limitation.

Preoperative and postoperative blood markers were measured at two prespecified time points: within 1 week before surgery and on postoperative day 1. Variables included CEA, CRP, albumin, white blood cell count, neutrophils, lymphocyte count, and platelets. SII was calculated as platelet count x neutrophil count/lymphocyte count, reflecting the balance between inflammatory cell activation and lymphocyte-associated immune competence. CAR was calculated as CRP/albumin, reflecting acute-phase inflammatory activation in relation to nutritional and systemic reserve. To reduce the influence of right-skewed distributions, SII and CAR were transformed using log1p(x) before modeling. POD1 postCAR was prespecified as an early perioperative host-response marker rather than a tumor-specific surrogate.

Adjuvant chemotherapy was coded as administered versus not administered from verified treatment records. Surgical approach was coded according to the operative record. MSI was coded as MSI-H versus MSS/MSI-L, and MMR was coded as dMMR versus pMMR according to routine pathology or molecular reports. MSI and MMR were not entered simultaneously into the same multivariable model because these variables were collinear in the verified data.

### Statistical analysis

Analyses were performed using R software (version 4.5.2). Continuous variables are presented as mean ± standard deviation or median (interquartile range), as appropriate, and categorical variables as number (percentage). Between-cohort comparisons were performed using the independent-samples t-test, Mann-Whitney U test, chi-square test, or Fisher’s exact test, as appropriate. Because patients with missing key predictors or incomplete follow-up were excluded before model development, the primary analyses were conducted on a complete-case basis; the study flow diagram summarizes these exclusions. Given the retrospective multicenter design, potential selection bias related to missingness cannot be excluded.

Candidate predictors were evaluated in the development cohort using a multistep strategy that combined clinical relevance with statistical selection. Univariable Cox proportional hazards regression was first used to assess associations with RFS. Variables with potential prognostic relevance were then entered into a least absolute shrinkage and selection operator (LASSO)-Cox model with 10-fold cross-validation, followed by assessment of multicollinearity using the variance inflation factor. Predictors retained after these steps were entered into multivariable Cox regression to derive the final model.

Continuous inflammatory biomarkers with skewed distributions, including preoperative systemic immune-inflammation index (preSII) and postoperative day-1 C-reactive protein-to-albumin ratio (postCAR), were transformed using log1p(x) before model fitting. Restricted cubic splines with three knots were used to evaluate nonlinearity. Effective model degrees of freedom were counted from the final Cox specification: six linear pathological/CEA terms, one log-transformed preSII term, and two spline terms for log-transformed postCAR, yielding 9 effective parameters. With 437 recurrence events in the development cohort, the final model had an events-per-variable ratio of 48.6.

To contextualize prognostic performance, two benchmark comparator models and one reduced final model were prespecified. The characteristics of the predefined comparator model frameworks are summarized in **Table 1**. Model A was a conventional clinicopathologic comparator including histological differentiation, pT stage, and pN stage. Model B was an expanded pathologic comparator that added EMVI, lymphovascular invasion, perineural invasion, tumor deposits, circumferential resection margin status, and preoperative CEA to Model A. Model C was then derived as the reduced final pathology-inflammation model after LASSO screening, collinearity assessment, nonlinear specification, and multivariable reduction; it retained histological differentiation, pN stage, EMVI, tumor budding, tumor deposits, preoperative CEA, log1p-transformed preSII, and spline-transformed log1p(postCAR). Accordingly, Model C should be interpreted as the clinically oriented reduced final model rather than as a strictly nested add-on extension of Model B. Discrimination was evaluated using Harrell’s C-index and time-dependent AUC, and calibration, decision curve analysis, reclassification, and risk-stratification performance were assessed as prespecified.

**Table 1:** Comparator and final model frameworks.

Model	Characteristics
Model A	Differentiation, pT, pN
Model B	Differentiation, pT, pN, EMVI, LVI, PNI, TD, CRM, preCEA
Model C (reduced final)	Differentiation, pN, EMVI, BD, TD, preCEA, log-preSII, spline log-postCAR

BD, tumor budding; CAR, C-reactive protein-to-albumin ratio; CRM, circumferential resection margin; EMVI, extramural venous invasion; LVI, lymphovascular invasion; PNI, perineural invasion; RCS, restricted cubic spline; SII, systemic immune-inflammation index; TD, tumor deposits. Note: Model A was the conventional clinicopathologic comparator, Model B the expanded pathologic comparator, and Model C the reduced final pathology-inflammation model derived after LASSO screening, collinearity assessment, nonlinear specification, and multivariable reduction.

Reviewer-requested sensitivity analyses were then performed by sequentially adding adjuvant chemotherapy, surgical approach, and MSI status to the final model. Internal validity was summarized with bootstrap optimism correction using 200 resamples. The development-derived risk cutoff was selected using the Youden index at the 3-year horizon among evaluable patients and then applied unchanged to the external validation cohort. Cutoff performance was reported separately in the development and external validation cohorts.

## Results

### Patient characteristics and outcome events

A total of 541 recurrence events were observed across the entire study population, including 437 of 1,008 patients (43.4%) in the development cohort and 104 of 252 patients (41.3%) in the external validation cohort.

In the verified revised datasets, MSI-H/dMMR status was present in 168 of 1,008 patients (16.7%) in the development cohort and 30 of 252 patients (11.9%) in the external validation cohort. Adjuvant chemotherapy was administered to 608 patients (60.3%) in the development cohort and 153 patients (60.7%) in the external validation cohort. The coded surgical approach distribution was also similar across cohorts, with category 1 recorded in 21 patients (2.1%) and 4 patients (1.6%), respectively (**Supplementary Table 4**).

The baseline clinicopathological characteristics of the development and external validation cohorts are summarized in **Table 2**. Overall, the two cohorts were broadly comparable. Demographic and laboratory variables were generally similar, with no significant differences in mean age (64.33 ± 12.07 vs. 63.27 ± 12.29 years; P = 0.218) or body mass index (23.38 ± 2.50 vs. 23.30 ± 2.64 kg/m²; P = 0.660). Most inflammatory and nutritional markers were also comparable between cohorts (all P > 0.05), although modest differences were observed in preoperative albumin and postoperative platelet count.

**Table 2:** Baseline characteristics of the development and external validation cohorts.

Characteristic	Development cohort (n=1008)	External validation cohort (n=252)	P value
Continuous variables			
Age, years	64.33 ± 12.07	63.27 ± 12.29	0.218
BMI	23.38 ± 2.50	23.30 ± 2.64	0.660
Preoperative ALB	40.62 ± 4.60	41.85 ± 4.37	<0.001
Preoperative PLT	212.77 ± 46.55	218.83 ± 48.68	0.075
Preoperative WBC	6.76 ± 2.07	6.62 ± 1.71	0.238
Preoperative NOS	4.15 ± 1.63	4.12 ± 1.31	0.793
Preoperative Lym	1.57 ± 0.61	1.60 ± 0.66	0.527
Preoperative SII	609.81 ± 288.63	633.99 ± 319.76	0.275
Postoperative CRP	54.87 ± 33.61	57.86 ± 37.66	0.251
Postoperative ALB	34.48 ± 3.76	33.99 ± 3.80	0.069
Postoperative CAR	1.64 ± 1.06	1.76 ± 1.25	0.147
Postoperative PLT	221.89 ± 37.52	212.96 ± 35.44	<0.001
Postoperative WBC	12.54 ± 4.15	12.53 ± 4.42	0.976
Postoperative NOS	8.92 ± 3.19	8.95 ± 3.35	0.886
Postoperative Lym	1.86 ± 0.82	1.97 ± 1.14	0.161
Postoperative SII	1148.46 ± 419.66	1134.50 ± 475.67	0.670
Categorical variables, n(%)			
Sex			0.030
Female	459 (45.5%)	95 (37.7%)	
Male	549 (54.5%)	157 (62.3%)	
Tumor location			0.045
Left colon	330 (32.7%)	82 (32.5%)	
Right colon	418 (41.5%)	87 (34.5%)	
Rectum	260 (25.8%)	83 (32.9%)	
Differentiation			<0.001
Well-differentiated	558 (55.4%)	170 (67.5%)	
Poorly differentiated	450 (44.6%)	82 (32.5%)	
pT stage			0.232
T1	172 (17.1%)	52 (20.6%)	
T2	238 (23.6%)	49 (19.4%)	
T3	286 (28.4%)	80 (31.7%)	
T4	312 (31.0%)	71 (28.2%)	
pN stage			0.068
N0	591 (58.6%)	166 (65.9%)	
N1	297 (29.5%)	66 (26.2%)	
N2	120 (11.9%)	20 (7.9%)	
AJCC stage			0.023
I	336 (33.3%)	82 (32.5%)	
II	255 (25.3%)	84 (33.3%)	
III	417 (41.4%)	86 (34.1%)	
EMVI			0.517
Negative	688 (68.3%)	166 (65.9%)	
Positive	320 (31.7%)	86 (34.1%)	
LVI			0.095
Negative	691 (68.6%)	187 (74.2%)	
Positive	317 (31.4%)	65 (25.8%)	
PNI			0.045
Negative	688 (68.3%)	189 (75.0%)	
Positive	320 (31.7%)	63 (25.0%)	
Tumor Budding			0.667
I	459 (45.5%)	118 (46.8%)	
II	269 (26.7%)	71 (28.2%)	
III	280 (27.8%)	63 (25.0%)	
Tumor Deposits			0.347
Negative	703 (69.7%)	184 (73.0%)	
Positive	305 (30.3%)	68 (27.0%)	
CRM			0.152
Negative	934 (92.7%)	226 (89.7%)	
Positive	74 (7.3%)	26 (10.3%)	
preCEA			<0.001
Negative	559 (55.5%)	174 (69.0%)	
Positive	449 (44.5%)	78 (31.0%)	

BMI, body mass index; CRP, C-reactive protein; ALB, albumin; PLT, platelet count; WBC, white blood cell count; NOS, neutrophils; Lym, lymphocytes; AJCC, American Joint Committee on Cancer; LVI, lymphovascular invasion; PNI, perineural invasion; Other abbreviations as in **Table 1**.

With respect to pathological characteristics, pT stage (P = 0.232) and pN stage (P = 0.068) were similar between cohorts, whereas AJCC stage (P = 0.023) and histological differentiation (P < 0.001) showed limited between-cohort variation. Importantly, major adverse pathological features, including LVI, PNI, EMVI, BD, TD, and CRM status, did not differ significantly between the two cohorts (all P > 0.05). These findings suggest that the external validation cohort was generally comparable to the development cohort while remaining independently assembled from other regional centers.

#### Variable selection and development of the reduced final pathology-inflammation model

To develop a parsimonious and clinically applicable prediction model for RFS, we applied a structured four-step variable selection strategy in the development cohort.

**Figure 2:**
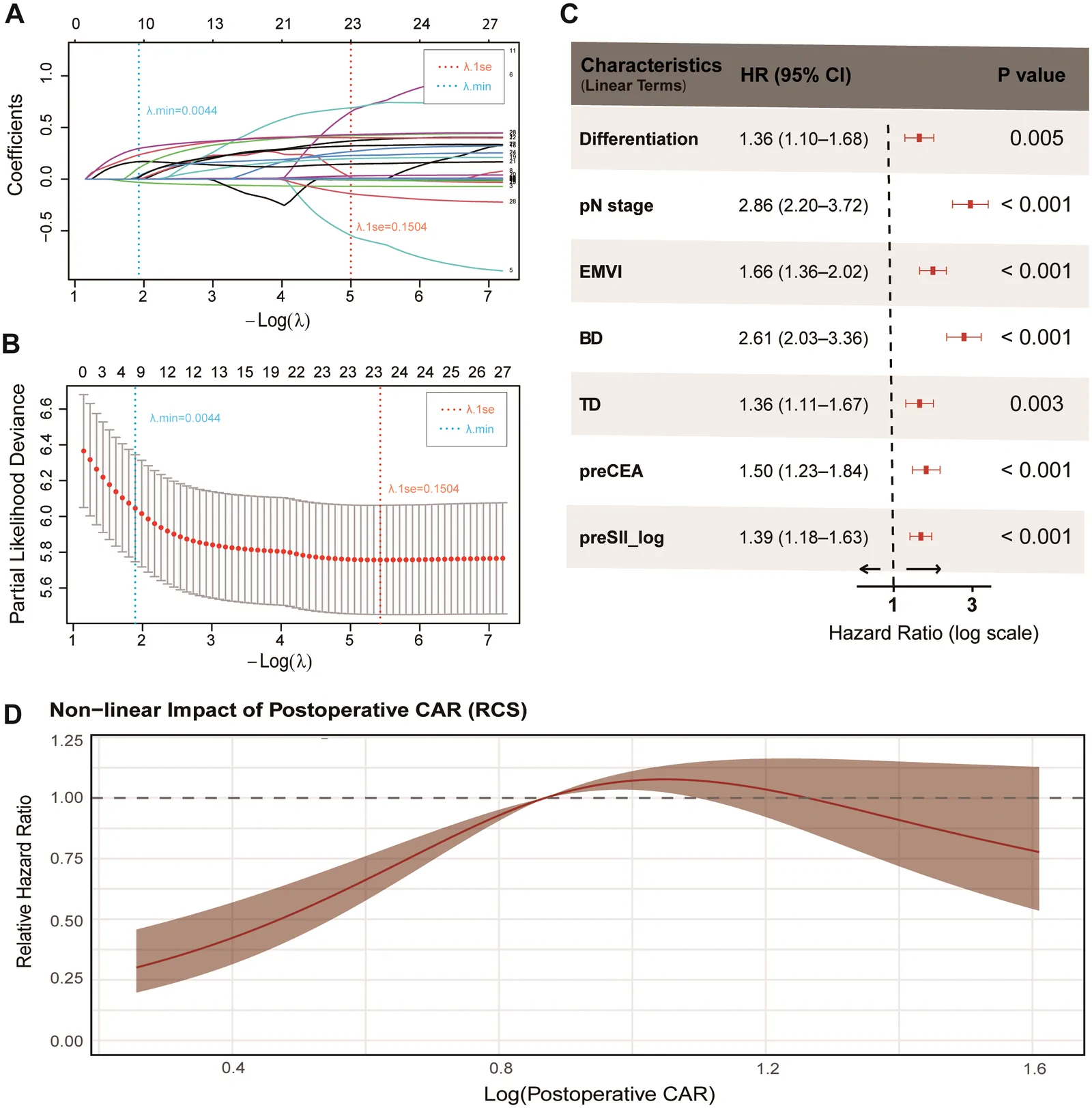
Variable selection and modeling procedures for the integrative pathology-inflammation model. **(A)** LASSO coefficient profiles of candidate predictors. **(B)** Ten-fold cross-validation for penalty selection. **(C)** Variance inflation factor assessment of candidate variables. **(D)** Restricted cubic spline analysis showing the nonlinear association between postoperative CAR and recurrence-free survival.

First, univariable Cox regression identified multiple candidate predictors associated with RFS (**Table 3**). Second, 10-fold cross-validated LASSO-Cox regression (minimum lambda = 0.0044; **Figures 2A, B**, **Supplementary Figure 1A**) retained 22 variables with nonzero coefficients. Although several individual blood-cell components were initially selected, composite inflammatory indices, particularly log-transformed preSII, were preferentially retained because they better summarize systemic inflammatory and immune-cell balance than isolated leukocyte counts. Third, variance inflation factor analysis showed that all retained variables had VIF values < 5, indicating no substantial multicollinearity (**Supplementary Figure 1B**). Variables such as AJCC stage, LVI, pT stage, and CRM status were not retained in the reduced final model after collinearity assessment and multivariable modeling because their prognostic information substantially overlapped with other selected features, particularly pN stage, EMVI, BD, and TD.

**Table 3:** Univariate and multivariate Cox regression analyses for recurrence-free survival.

Characteristics	Univariate HR (95% CI)	P-value	Multivariate HR (95% CI)	P-value
Continuous variables				
Age, years	1.01 (0.99–1.01)	0.105	–	–
BMI	0.98 (0.94–1.01)	0.229	–	–
Preoperative Albumin	0.87 (0.85–0.89)	<0.05	–	–
Preoperative Platelet	1.00 (1.00–1.00)	0.17	–	–
Preoperative WBC	1.09 (1.05–1.14)	<0.05	–	–
Preoperative Neutrophil	1.21 (1.15–1.27)	<0.05	–	–
Preoperative Lymphocyte	0.74 (0.63–0.86)	<0.05	–	–
Preoperative SII	3.02 (2.45–3.72)	<0.05	1.67 (1.30–2.13)	<0.05
Postoperative CRP	1.01 (1.00–1.01)	<0.05	–	–
Postoperative Albumin	0.92 (0.90–0.95)	<0.05	–	–
Postoperative CAR	2.17 (1.73–2.72)	<0.05	*Nonlinear*	<0.05
Postoperative Platelet	1.01 (1.01–1.01)	<0.05	–	–
Postoperative WBC	1.03 (1.01–1.05)	0.014	–	–
Postoperative Neutrophil	1.04 (1.01–1.07)	0.009	–	–
Postoperative Lymphocyte	1.00 (0.89–1.12)	0.978	–	–
Postoperative SII	2.39 (1.84–3.10)	<0.05	–	–
Categorical variables				
Sex	0.99 (0.82–1.19)	0.877	–	–
Tumor location	1.05 (0.93–1.19)	0.413	–	–
Differentiation	2.62 (2.16–3.19)	<0.05	1.36 (1.10–1.68)	0.005
pT stage	1.56 (1.42–1.72)	<0.05	–	–
pN stage	2.46 (2.19–2.76)	<0.05	1.69 (1.49–1.93)	<0.05
AJCC stage	2.74 (2.39–3.15)	<0.05	–	–
EMVI	2.80 (2.32–3.38)	<0.05	1.66 (1.37–2.01)	<0.05
LVI	2.60 (2.15–3.14)	<0.05	–	–
PNI	2.89 (2.39–3.49)	<0.05	–	–
Tumor budding	2.35 (2.10–2.64)	<0.05	1.61 (1.43–1.82)	<0.05
Tumor deposits	2.59 (2.15–3.13)	<0.05	1.36 (1.11–1.67)	<0.05
CRM status	1.71 (1.27–2.31)	<0.05	–	–
Preoperative CEA	2.46 (2.02–2.98)	<0.05	1.50 (1.23–1.84)	<0.05

Abbreviations as in **Table 1** or **Table 2**.

Finally, restricted cubic splines with three knots were used to assess the functional form of continuous variables. Log-transformed postCAR showed a significant nonlinear association with RFS (P < 0.05) and was therefore modeled flexibly using restricted cubic splines. In the final multivariable Cox model (**Figure 2C**), eight variables remained independently associated with RFS: histological differentiation, pN stage, EMVI, BD, TD, preoperative CEA, log-transformed preSII, and spline-modeled log-transformed postCAR (**Figure 1D**). These retained variables jointly represent two complementary dimensions of recurrence risk: pathological invasiveness and perioperative inflammatory host response. A pT-inclusive sensitivity specification showed near-identical discrimination, supporting the interpretation that pT carried limited independent incremental information once the other high-risk pathological features were incorporated (**Supplementary Table 1**, **2**).

#### Discriminative performance and reclassification of the reduced final pathology-inflammation model

To contextualize the prognostic contribution of perioperative inflammatory information, we compared the reduced final pathology-inflammation model with the two prespecified benchmark comparator models.

**Figure 3:**
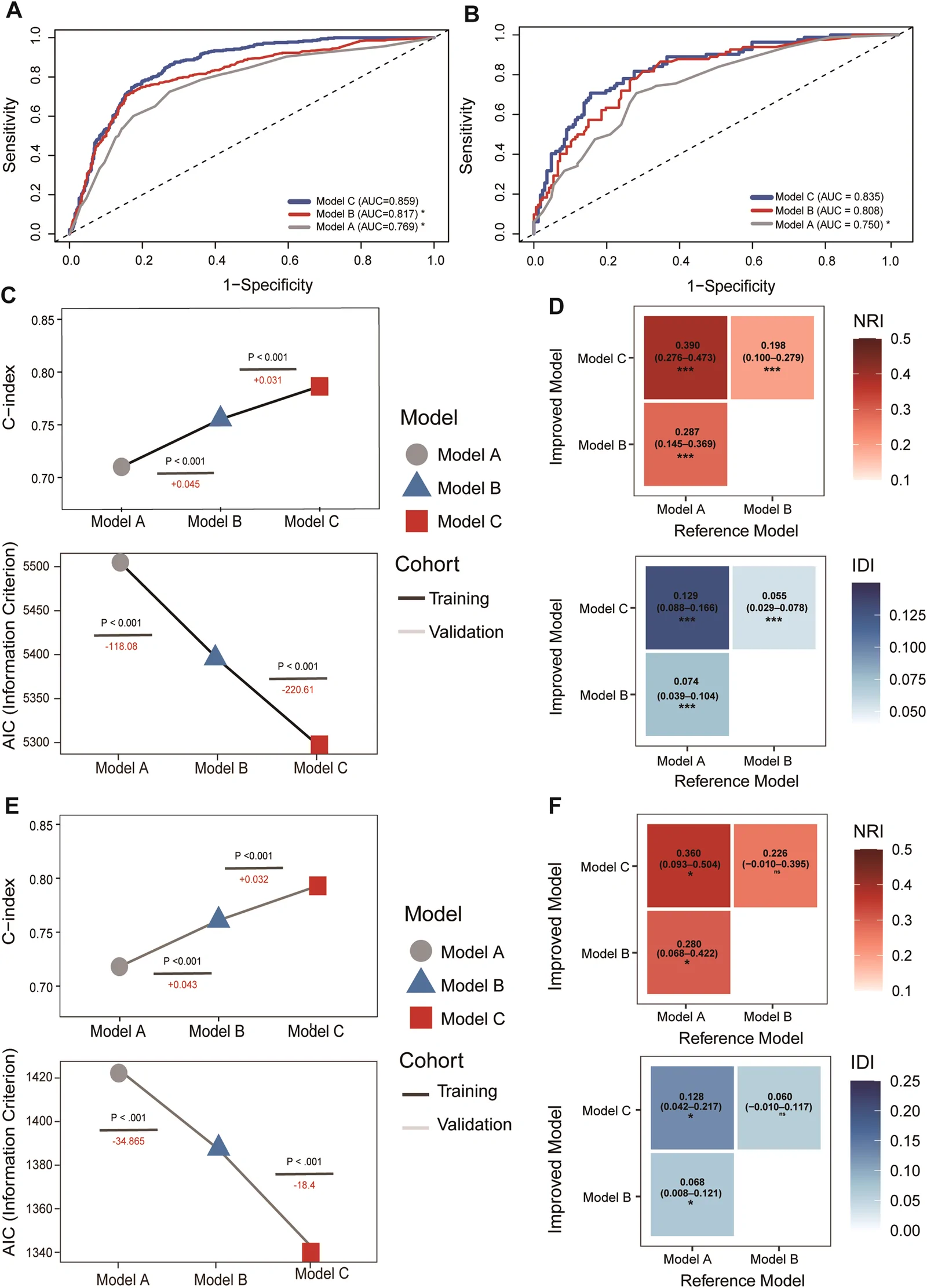
Comparative discriminative performance of the model. ROC curves for the 3-year development **(A)** and external validation **(B)** cohorts. Comparison of C-index and AIC across models in the development **(C)** and external validation **(E)** cohorts. Reclassification metrics (NRI and IDI) for model comparisons in the training **(D)** and validation **(F)** cohorts.

The reduced final pathology-inflammation model showed the best discriminative performance in both cohorts. In the development cohort, the 3-year time-dependent AUC was 0.859 for the reduced final model, compared with 0.817 for the pathology-enhanced model and 0.789 for the reference clinicopathologic model (**Figure 3A**). In the external validation cohort, the corresponding AUCs were 0.835, 0.808, and 0.750, respectively (**Figure 3B**). Similar findings were observed at 2 and 5 years (**Supplementary Figures 2A–D**). The DCA curves of the three models are presented in Supplementary **Figures 2E–J**. C-index analysis showed the same ranking across cohorts (all pairwise P < 0.001; **Figures 2C, E**), whereas the reduced final model also yielded the lowest AIC values, indicating the best overall model fit among the three models. Because Model C was also re-specified through variable reduction and nonlinear modeling, its superior performance should be interpreted as the combined effect of optimized model composition and incorporation of perioperative inflammatory markers rather than as a pure add-on effect of inflammation alone.

Reclassification analyses supported the incremental value of the reduced final pathology-inflammation model beyond the prespecified comparator models. In the development cohort (**Figure 3D**) and the validation cohort (**Figure 3F**), compared with the conventional clinicopathologic Model A, the reduced final Model C achieved an NRI of 0.360 (95% CI, 0.093-0.504) and an IDI of 0.128 (95% CI, 0.042-0.217). Compared with the pathology-enhanced Model B, Model C achieved an NRI of 0.226 (95% CI, -0.010 to 0.395) and an IDI of 0.060 (95% CI, -0.010 to 0.117). Compared with the pathology-enhanced Model B, Model C achieved an NRI of 0.226 (95% CI, -0.010 to 0.395) and an IDI of 0.060 (95% CI, -0.010 to 0.117). Because the Model C versus Model B reclassification confidence intervals crossed the null, this comparison was interpreted as directionally supportive rather than definitive.

### Calibration, clinical utility, and risk stratification

**Figure 4:**
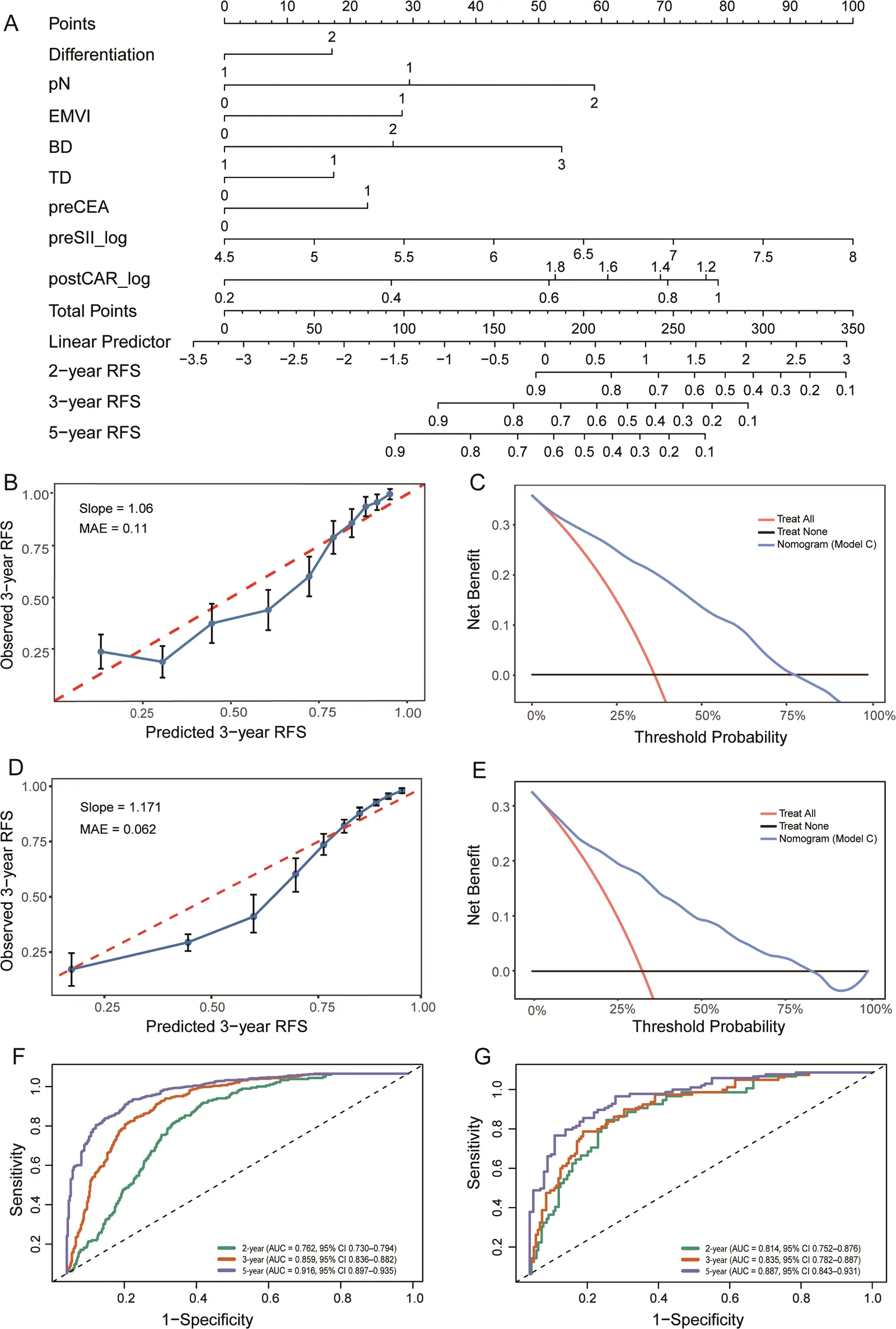
Calibration, clinical utility, and nomogram-based risk stratification of the integrative pathology-inflammation model. **(A)** Nomogram for 2-, 3-, and 5-year RFS incorporating the eight retained predictors. **(B, D)** Calibration curves in the development and external validation cohorts. **(C, E)** Decisioncurve analyses in the development and external validation cohorts. **(F, G)** Time-dependent ROC curves for 2-, 3-, and 5-year RFS prediction in the training and external validation cohorts.

The reduced final multivariable Cox model was translated into a nomogram incorporating the eight retained predictors (**Figure 4A**).

The final model used 9 effective parameters and 437 recurrence events, yielding an events-per-variable ratio of 48.6. Bootstrap-based internal validation showed limited optimism: the apparent Harrell C-index was 0.786, mean bootstrap optimism was 0.003, and the optimism-corrected C-index was 0.783. In the external validation cohort, the final model Harrell C-index was 0.793. Sequentially adding adjuvant chemotherapy, surgical approach, and MSI status did not materially change discrimination (external validation C-index, 0.793 for all extended models; **Supplementary Table 6**).

Calibration plots showed good agreement between predicted and observed RFS probabilities. For 3-year RFS, the calibration slope was 1.06 (MAE = 0.11) in the development cohort (**Figure 4B**) and 1.17 (MAE = 0.062) in the external validation cohort (**Figure 4D**). Calibration at 2 and 5 years was similarly acceptable in both cohorts (**Supplementary Figures 3A–D**). Time-dependent ROC curves of the nomogram (**Figures 4F, G**) were consistent with the discrimination results described above, indicating that the pathology-inflammation framework provided stable individualized recurrence-risk estimates across clinically relevant time horizons.

Decision curve analysis indicated that the nomogram provided greater net benefit than either treat-all or treat-none strategies across a broad range of threshold probabilities in both the development cohort (**Figure 4C**) and the external validation cohort (**Figure 4E**). Similar patterns were observed at 2 and 5 years (**Supplementary Figures 3E–H**), suggesting that inflammatory host-response information may help refine postoperative surveillance decisions when combined with established pathological risk factors.

**Figure 5:**
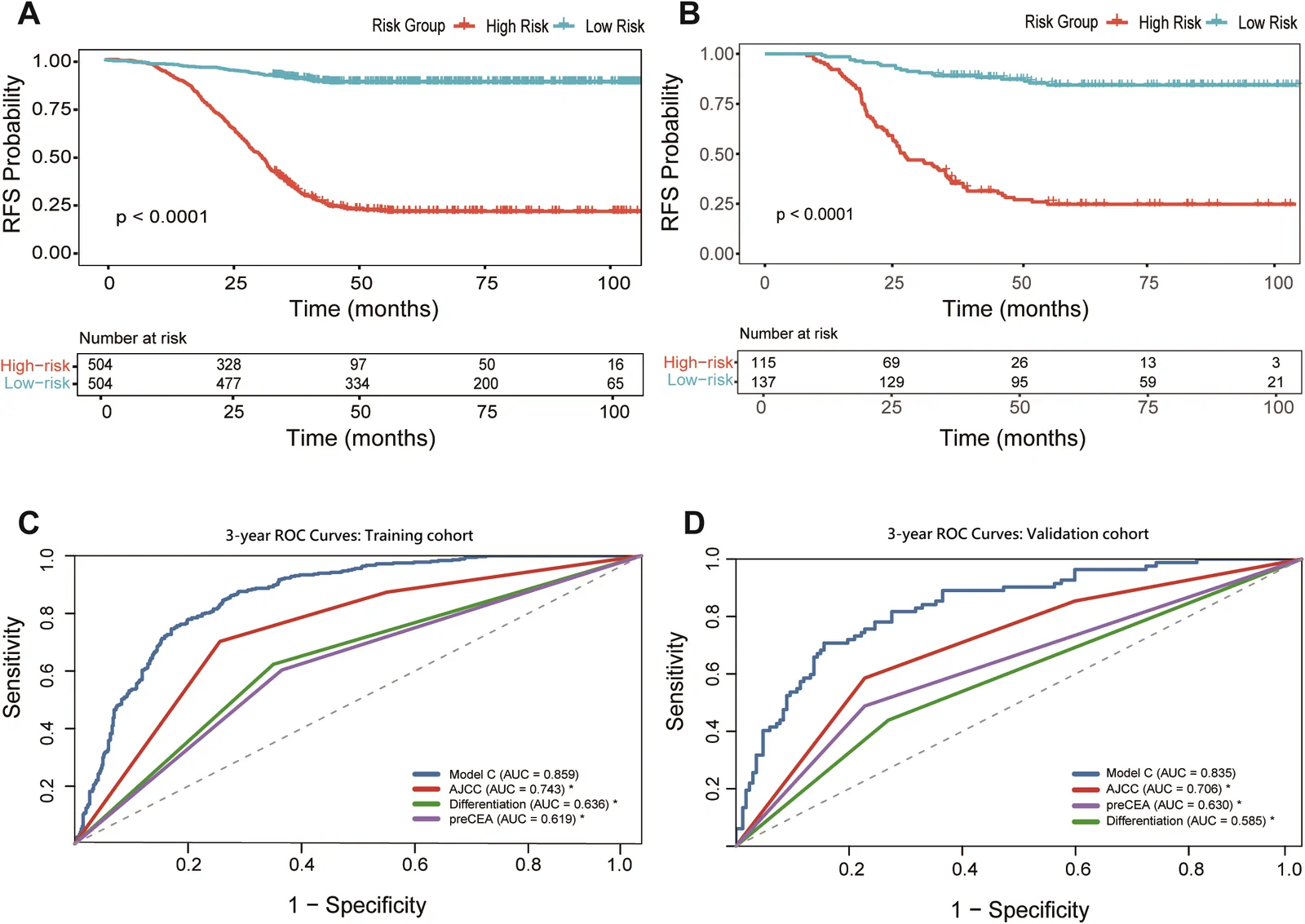
Survival stratification and discriminative ability of the integrative pathology-inflammation model. Kaplan-Meier curves of recurrence-free survival for high- and low-risk groups in the development **(A)** and external validation **(B)** cohorts.

Using the optimal cutoff derived from the reduced final pathology-inflammation model, patients were stratified into high-risk and low-risk groups. Kaplan-Meier analysis demonstrated clear separation of RFS curves in both cohorts (log-rank P < 0.0001; **Figures 5A, B**). Patients in the high-risk group consistently showed poorer RFS throughout follow-up, supporting the model’s ability to identify individuals in whom tumor invasiveness and systemic inflammatory burden converge to produce a higher recurrence-risk phenotype. **Figures 5C** and **5D** demonstrate that Model C possesses superior discriminatory power compared to individual tumor pathology and tumor markers.

At the 3-year horizon, the development-derived cutoff showed sensitivity of 86.2% and specificity of 72.6% in the development cohort. When the same cutoff was applied unchanged to the external validation cohort, sensitivity was 78.0%, specificity was 72.5%, PPV was 58.2%, and NPV was 87.1%; Kaplan-Meier separation remained significant (log-rank P < 0.001; **Supplementary Table 8**). These results indicate that the cutoff retained useful discrimination externally but should still be recalibrated prospectively before clinical deployment. 

## Discussion

In this multicenter retrospective study with external validation across three additional regional hospitals, we developed and evaluated an integrative pathology-inflammation model for predicting postoperative recurrence after curative resection of non-metastatic CRC. The model incorporated both pathological indicators of tumor aggressiveness and perioperative inflammatory biomarkers, and consistently outperformed the comparator clinicopathologic models in terms of discrimination, calibration, and clinical net benefit. These findings suggest that recurrence risk after curative surgery may be more accurately characterized by combining tumor-invasive phenotype with measurable host inflammatory response, rather than relying on stage-based assessment alone (20, 21). The findings therefore support preliminary multicenter transportability within a regional Chinese clinical setting.

The pathological variables retained in the reduced final model are biologically and clinically plausible. EMVI, tumor budding, and tumor deposits represent distinct yet complementary dimensions of local invasiveness, vascular dissemination, and metastatic potential, and each has been associated with adverse oncologic outcomes in CRC (22–24). pT stage remains a cornerstone of anatomic staging (25), and it should not be regarded as unimportant in this disease context. Rather, in the present dataset, a substantial part of its prognostic contribution appeared to overlap with nodal burden and other high-risk microscopic pathological features. Consistent with this interpretation, the pT-inclusive sensitivity model showed nearly identical discrimination to the reduced final model, with only modest differences in likelihood-based fit (**Supplementary Table 1**, **2**). We therefore interpret pT as having limited independent incremental contribution after joint adjustment for pN stage, EMVI, BD, and TD, while preserving the pT-inclusive specification as a clinically face-valid sensitivity analysis.

A key feature of the present model is the integration of perioperative inflammatory burden. PreSII reflects the preoperative balance among platelets, neutrophils, and lymphocytes, thereby summarizing pro-inflammatory activation and lymphocyte-mediated host response. PostCAR, however, requires more cautious interpretation. Although postoperative day-1 CRP and albumin may capture early host-response biology, they can also be influenced by surgical trauma, operative complexity, perioperative infection, transfusion, albumin replacement, and early postoperative recovery. In the revised analysis, adjustment for surgical approach did not materially attenuate model performance, but more granular perioperative variables were unavailable; therefore, postCAR should be interpreted as an early postoperative risk marker rather than a tumor-specific biomarker.

The comparison across the model frameworks helps contextualize the incremental value of the final pathology-inflammation model. Several recent colorectal cancer recurrence or survival prediction models have incorporated molecular, nodal, longitudinal serum-marker, and stage-specific clinicopathological features (26–31). Prior studies have also shown that clinicopathological variables and inflammatory biomarkers are prognostically relevant in colorectal and rectal cancer, including the recent Medicine study by Şentürk and Harmantepe (37). The novelty of the present work is therefore not the isolated prognostic effect of any single inflammatory marker, but the integration of invasive pathological features, preoperative inflammatory burden, and early postoperative inflammatory response in a multicenter externally validated recurrence model. Even so, Model C was derived after shrinkage, collinearity assessment, nonlinear specification, and multivariable reduction; therefore, apparent gains over comparator models may remain optimistic and require prospective confirmation.

From a biological perspective, the combined inclusion of BD, TD, EMVI, preSII, and postCAR is biologically plausible as a marker set spanning tumor invasiveness and host-response status. Tumor budding has been linked to epithelial-mesenchymal transition and stem cell-like phenotypes (32, 33), whereas perioperative inflammatory activation may be consistent with immune suppression, angiogenic remodeling, and outgrowth of minimal residual disease in susceptible patients (34, 35). Neutrophil- and platelet-dominant inflammation may promote tumor cell adhesion and metastatic niche formation, while reduced lymphocyte-related immune competence may impair antitumor surveillance. Even so, the present study was designed as a clinical prediction analysis rather than a mechanistic investigation. Accordingly, these observations should be interpreted as biologically plausible rather than as direct evidence of causal pathways.

These findings may have implications for postoperative management. The model is not intended to replace guideline-based follow-up, but rather to complement existing surveillance strategies by identifying patients at increased risk of recurrence after curative surgery. Current follow-up recommendations, including those from NCCN and ESMO, are largely stage-based and broadly applicable, but they have limited granularity for individualized risk estimation (36). In this context, patients classified as high risk may warrant closer surveillance or more intensive monitoring within multidisciplinary care pathways, whereas those classified as low risk may remain suitable for standard follow-up. Any such use should be integrated with stage, comorbidity, and treatment information rather than applied in isolation. The model may also provide a practical platform for future studies that combine routine inflammatory markers with immune-contexture assessment, ctDNA-guided follow-up, or biomarker-guided management strategies.

The newly incorporated treatment and molecular-marker analyses also inform interpretation. Adjuvant chemotherapy was administered to approximately 60% of patients in both cohorts, and adding this variable did not materially change model discrimination. MSI-H/dMMR status was present in 16.7% of the development cohort and 11.9% of the validation cohort; adding MSI status likewise did not materially change the model C-index. These analyses support the robustness of the pathology-inflammation model, but they do not establish causal treatment effects because treatment regimen, dose intensity, completion, and time to treatment initiation were not uniformly available. This simplified treatment coding should therefore be interpreted alongside guideline-based, stage-adapted adjuvant therapy recommendations (38).

Several limitations should be acknowledged. First, despite the multicenter design and external validation, this was a retrospective study and is therefore subject to selection bias and residual confounding. Second, the development cohort was derived from a single high-volume center, whereas the external validation cohort combined three regional hospitals. Harmonized definitions and centralized data checks were used, but differences in pathology interpretation, laboratory platforms, perioperative management, adjuvant treatment delivery, and follow-up practice may still have influenced recurrence ascertainment and model performance. Third, no centralized pathology rereview was performed; therefore, variables such as EMVI, tumor budding, and tumor deposits may retain inter-pathologist variability. Fourth, postoperative day-1 CAR may reflect surgical inflammation and early postoperative care in addition to tumor-host biology. Fifth, adjuvant chemotherapy was incorporated as administered versus not administered, but regimen, completion, dose intensity, and time to initiation were unavailable. Sixth, MSI/MMR status was added in the revised analysis, but RAS/BRAF mutation status and ctDNA were not available; this limits biological interpretation and comparison with contemporary molecular risk models. Seventh, although calibration, decision curve analysis, cutoff validation, and bootstrap optimism correction supported model performance, these analyses do not establish routine clinical utility. Prospective validation, center-level recalibration, and impact analysis are required before clinical implementation.

In summary, the present study supports the value of integrating pathological invasiveness with perioperative systemic inflammation for postoperative recurrence assessment in stage I-III CRC. The revised analyses show that the model retained stable performance after adjustment for adjuvant chemotherapy, surgical approach, and MSI status, while also clarifying the need for cautious interpretation of postoperative inflammatory markers and prospective validation before clinical use.

## Conclusion

An integrative pathology-inflammation model combining pathological aggressiveness with perioperative systemic inflammatory burden improved postoperative recurrence prediction in CRC and maintained its performance in an external multicenter validation cohort and in sensitivity analyses incorporating treatment, surgical approach, and MSI/MMR status. The model may support postoperative risk stratification after further prospective validation and center-specific calibration, but it should not yet be used as a stand-alone basis for routine clinical decision-making.

## Data Availability

The raw data supporting the conclusions of this article will be made available by the authors, without undue reservation.

## Supplementary material

Supplementary Figure 1 Variable Selection and Multicollinearity Assessment. **(A)** LASSO coefficient profiles showing variables retained at the optimal λ; LASSO regression identified the variables with the strongest prognostic contribution by shrinking less informative coefficients toward zero. **(B)** Variance inflation factor (VIF) analysis evaluating multicollinearity among candidate predictors; all key variables showed VIF values below the common threshold of 10, indicating no substantial multicollinearity.

Supplementary Figure 2 Discriminative performance of the benchmark comparator models (Model A and Model B) and the reduced final pathology-inflammation model (Model C). **(A–D)** ROC curves for 2-year **(A, B)** and 5-year **(C, D)** recurrence-free survival in the development **(A, C)** and external validation (B, D) cohorts.

Supplementary Figure 3 Calibration and clinical utility of the reduced final pathology-inflammation model (Model C) for predicting recurrence-free survival. **(A–D)** Development **(A, C)** and external validation **(B, D)** calibration curves for 2-year **(A, B)** and 5-year **(C, D)** recurrence-free survival. **(E–H)** Decision curve analyses at 2 and 5 years in the development and external validation cohorts.
