# Supplementary material for: Integrating perioperative inflammation and pathological invasiveness to predict recurrence after curative colorectal cancer surgery: a multicenter cohort study
Source: Front Oncol. 2026 Aug 12;16:1861588. doi: 10.3389/fonc.2026.1861588 (PMC13506383; doi:10.3389/fonc.2026.1861588)
Supplement: Supplementary file 1 [file Table1.docx]

**Supplementary Tables**

**Table S1. Coefficient Redistribution After Excluding pT From the pT-Inclusive Sensitivity Model**

| Variable | Coef (pT-inclusive model) | Coef (reduced final model) | **Change (Δ)** |
| --- | --- | --- | --- |
| pT | 0.1493 | - | - |
| pN | 0.4683 | 0.526 | 0.0577 |
| BD | 0.4689 | 0.4794 | 0.0105 |
| EMVI | 0.4921 | 0.505 | 0.0129 |
| TD | 0.303 | 0.3106 | 0.0076 |
| Differentiation | 0.2681 | 0.3059 | 0.0378 |
| preCEA | 0.4048 | 0.4066 | 0.0018 |
| Log-transformed preSII | 0.5111 | 0.5105 | -0.0006 |
| Spline-modeled log-transformed postCAR | 2.4123 | 2.373 | -0.0393 |

**Table S2. pT-Inclusive Sensitivity Model Versus Reduced Final Model**

| Metrics | pT-inclusive model | Reduced final model | **Difference** |
| --- | --- | --- | --- |
| Degrees of Freedom | 10 | 9 | -1 |
| Likelihood Ratio Chi2 | 520.66 | 512.82 | -7.84 |
| Nagelkerke R2 | 0.405 | 0.4 | -0.005 |
| Dxy (Somers' D) | 0.573 | 0.572 | -0.001 |
| C-index | 0.7865 | 0.786 | -0.0005 |
| AIC | 5295.05 | 5300.9 | 5.85 |
| AUC | 0.860 | 0.859 | -0.001 |

**Table S3. Performance of the Reduced Final Model Across Cohorts**

| **Variable** | **Time Point** | **Cutoff** | **Sensitivi-ty (%)** | **Specific-ity (%)** | **PPV (%)** | **NPV (%)** | **Accuracy (%)** | **Youden**  **Index** | **AUC** |
| --- | --- | --- | --- | --- | --- | --- | --- | --- | --- |
| Development cohort | 2-year | -0.068 | 85.5 | 60.1 | 32.6 | 94.8 | 64.8 | 0.456 | 0.762 |
|  | 3-year | -0.111 | 87.6 | 71.1 | 64.5 | 90.5 | 77.3 | 0.587 | 0.859 |
|  | 5-year | -0.101 | 87 | 80.7 | 84.8 | 83.3 | 84.2 | 0.677 | 0.916 |
| External validation cohort | 2-year | 0.204 | 76.5 | 77.6 | 46.4 | 92.9 | 77.4 | 0.541 | 0.814 |
|  | 3-year | 0.204 | 70.7 | 84.4 | 69 | 85.5 | 79.9 | 0.552 | 0.835 |
|  | 5-year | -0.346 | 88.5 | 75 | 79.3 | 85.7 | 82 | 0.635 | 0.888 |

## Table S4. Newly incorporated treatment and molecular variables

| **Cohort** | **Variable** | **Level** | **n** | **%** |
| --- | --- | --- | --- | --- |
| Development | MSI | MSI-H | 168 | 16.700 |
| Development | MSI | MSS/MSI-L | 840 | 83.300 |
| Development | MMR | dMMR | 168 | 16.700 |
| Development | MMR | pMMR | 840 | 83.300 |
| Development | Adjuvant chemotherapy | 0 | 400 | 39.700 |
| Development | Adjuvant chemotherapy | 1 | 608 | 60.300 |
| Development | Surgical approach | 0 | 987 | 97.900 |
| Development | Surgical approach | 1 | 21 | 2.100 |
| External validation | MSI | MSI-H | 30 | 11.900 |
| External validation | MSI | MSS/MSI-L | 222 | 88.100 |
| External validation | MMR | dMMR | 30 | 11.900 |
| External validation | MMR | pMMR | 222 | 88.100 |
| External validation | Adjuvant chemotherapy | 0 | 99 | 39.300 |
| External validation | Adjuvant chemotherapy | 1 | 153 | 60.700 |
| External validation | Surgical approach | 0 | 248 | 98.400 |
| External validation | Surgical approach | 1 | 4 | 1.600 |

## Table S5. Missing-data rates for analytic variables

| **Cohort** | **Variable** | **Missing n** | **Missing %** |
| --- | --- | --- | --- |
| Development | RFS | 0 | 0 |
| Development | RFStime | 0 | 0 |
| Development | Differentiation | 0 | 0 |
| Development | pN stage | 0 | 0 |
| Development | EMVI | 0 | 0 |
| Development | Tumor budding | 0 | 0 |
| Development | Tumor deposits | 0 | 0 |
| Development | Preoperative CEA | 0 | 0 |
| Development | Preoperative SII | 0 | 0 |
| Development | Postoperative CAR | 0 | 0 |
| Development | MSI | 0 | 0 |
| Development | MMR | 0 | 0 |
| Development | Adjuvant chemotherapy | 0 | 0 |
| Development | Surgical approach | 0 | 0 |
| External validation | RFS | 0 | 0 |
| External validation | RFStime | 0 | 0 |
| External validation | Differentiation | 0 | 0 |
| External validation | pN stage | 0 | 0 |
| External validation | EMVI | 0 | 0 |
| External validation | Tumor budding | 0 | 0 |
| External validation | Tumor deposits | 0 | 0 |
| External validation | Preoperative CEA | 0 | 0 |
| External validation | Preoperative SII | 0 | 0 |
| External validation | Postoperative CAR | 0 | 0 |
| External validation | MSI | 0 | 0 |
| External validation | MMR | 0 | 0 |
| External validation | Adjuvant chemotherapy | 0 | 0 |
| External validation | Surgical approach | 0 | 0 |

## Table S6. Sequentially adjusted model performance

| **Model** | **Parameters** | **Development events** | **EPV** | **Partial log-likelihood** | **AIC** | **Development C-index** | **External validation C-index** |
| --- | --- | --- | --- | --- | --- | --- | --- |
| Final model | 9 | 437 | 48.600 | -2641.6 | 5301.2 | 0.786 | 0.793 |
| Final + adjuvant chemotherapy | 10 | 437 | 43.700 | -2640.9 | 5301.9 | 0.786 | 0.793 |
| Final + chemotherapy + surgical approach | 11 | 437 | 39.700 | -2640.9 | 5303.9 | 0.786 | 0.793 |
| Final + chemotherapy + surgical approach + MSI | 12 | 437 | 36.400 | -2640.9 | 5305.9 | 0.786 | 0.793 |

## Table S7. Bootstrap internal validation and EPV

| **Bootstrap repetitions requested** | **Successful repetitions** | **Failed repetitions** | **Apparent C-index** | **Mean optimism** | **Optimism-corrected C-index** | **Final model effective df** | **Events-per-variable** |
| --- | --- | --- | --- | --- | --- | --- | --- |
| 200 | 200 | 0 | 0.786 | 0.003 | 0.783 | 9 | 48.600 |

## Table S8. Development-derived cutoff and external validation

| **Cohort** | **cutoff** | **evaluable_n** | **events_by_36m** | **high_risk_n** | **sensitivity** | **specificity** | **PPV** | **NPV** | **Youden** | **Log-rank chi-square** | **Log-rank P** |
| --- | --- | --- | --- | --- | --- | --- | --- | --- | --- | --- | --- |
| Development | -0.070 | 968 | 363 | 479 | 0.862 | 0.726 | 0.653 | 0.898 | 0.588 | 467.1 | <0.001 |
| External validation | -0.070 | 249 | 82 | 110 | 0.780 | 0.725 | 0.582 | 0.871 | 0.505 | 94.860 | <0.001 |
